# Living With COVID-19: A Systemic and Multi-Criteria Approach to Enact Evidence-Based Health Policy

**DOI:** 10.3389/fpubh.2020.00294

**Published:** 2020-06-16

**Authors:** Didier Raboisson, Guillaume Lhermie

**Affiliations:** IHAP, University of Toulouse, INRA, ENVT, Toulouse, France

**Keywords:** COVID-19, population medicine, systemic approach, evidence-based policy, social-ecological system (SES)

## Abstract

The lifting of COVID-19 (coronavirus disease 2019) lockdown requires, in the short and medium terms, a holistic and evidence-based approach to population health management based on combining risk factors and bio-economic outcomes, including actors' behaviors. This dynamic and global approach to health control is necessary to deal with the new paradigm of living with an infectious disease, which disrupts our individual freedom and behaviors. The challenge for policymakers consists of defining methods of lockdown-lifting and follow-up (middle-term rules) that best meet the needs for resumption of economic activity, societal wellbeing, and containment of the outbreak. There is no simple and ready-to-use way to do this since it means considering several competing objectives at the same time and continuously adapting the strategy and rules, ideally at local scale. We propose a framework for creating a precision evidence-based health policy that simultaneously considers public health, economic, and societal dimensions while accounting for constraints and uncertainty. It is based on the four following principles: integrating multiple and heterogeneous information, accepting navigation with uncertainty, adjusting the strategy dynamically with feedback mechanisms, and managing clusters through a multi-scalar conception. The evidence-based policy intervention for COVID-19 obtained includes scientific background via epidemiological modeling and bio-economic modeling. A set of quantitative and qualitative indicators are used as feedback to precisely monitor the societal-economic-epidemiological dynamics, allowing tightening or loosening of measures before epidemic damage (re-)occurs. Altogether, this allows an evidence-based policy that steers the strategy with precision and avoids any political shock.

## Introduction

The coronavirus disease 2019 (COVID-19) pandemic has been perceived as a major, unprecedented public health threat sparing no country with a speed of onset that has lead policymakers worldwide to implement drastic control measures very quickly (1). The first objective was to avoid a massive mortality burden, which led to extensive lockdowns to contain the dissemination of the outbreak. As days pass, lockdowns prove effective in limiting public health damage, while, at the same time, social movements rise to advocate for freedom to work and circulate (2). Indeed, COVID-19 represents a change in paradigm for our society and the healthcare system. In the last few decades, outbreaks have been maintained locally and have been limited over time, which makes COVID-19 a novel entity (3). The management of infectious disease can follow two alternative strategies: the first one is to eradicate the disease, and the second one is to learn to live with the disease and mitigate its impact. As of mid-2020, eradicating a disease such as COVID-19 seems not to be an option: vaccines are not available, protective immunity after infection is challenged, and immunity duration is unknown; moreover, quick development of herd immunity would likely come at high public health costs, with a significant number of deaths and a large healthcare expenditure.

Living with COVID-19 will lead to substantial changes in individual freedom and behaviors and directly change medical, economic, societal, and political stakes worldwide. The very challenge for policymakers consists of implementing a sustainable approach for the economic and social sector, which will require the lifting of restrictions sooner or later. The ultimate goal of lockdown-lifting is to mitigate the impact on the country's economy and on the well-being of individuals while containing the spread of the outbreak in a way that is manageable for the healthcare services, without having to face ethical dilemmas such as equity in healthcare or additional risks of death in case of infection. The optimal lockdown-lifting method would be the one that best meets these four objectives (mitigating the spread of the outbreak, maintaining economic activity, and social well-being, and ensuring political stability) in both the short and the long terms.

This crisis reveals the difficulty of implementing responses to seemingly simple problems (a single infectious agent) but which are actually fundamentally complex and gaining acceptance of them by citizens. This observation is not new in the public health literature, and academics, as well as some institutions, invite policymakers to pledge policies accounting for multiple parameters. Additionally, political scientists have analyzed agenda-setting in light of the interdependence of people acting in a political and institutional context (4, 5). In the case of COVID-19, a context of emergency leads to the envisioning of responses articulating (i) biological and economic constraints, including the behavior of individuals, and (ii) high biologic and economic uncertainty. These facts lead to the seeking of original solutions that are able to handle multiple criteria simultaneously and are sufficiently acceptable by individuals (for their own safety and for compliance with rules for collective purposes). A holistic approach to health management, beyond outbreak management, is therefore necessary (6). It should dynamically handle multiple risk factors and multiple economic and biologic outcomes and be customized at various geographical scales. Such an approach must combine medicine, epidemiology, and economics and differs from normal epidemiological approaches centered on an infectious agent or a syndrome.

We propose to lay down the characteristics of a holistic approach, accounting for several objectives and different time steps, that is required to manage lockdown-lifting and the COVID-19 endemic situation.

To do so, we rely on social-ecological approaches developed in the field of environmental economics and in public health policy. In Ostrom's “diagnostic approach,” the Social-Ecological System (SES) framework was designed to address coordination problems of natural resource management and help prioritize the most relevant variables (7, 8). SESs are complex adaptive systems with many locally interacting components evolving with non-linear dynamics, sometimes unpredictably (9, 10). Adapted to the current COVID-19 situation, the SES relates outcomes such as health, well-being, and economic welfare to interactions between humans, e.g., number of contacts or conflicts among people, which are influenced by the resource system, the governance system, and users in a given social, economic, and political setting.

In parallel, a significant amount of literature advocates for systemic approaches in public health (11). One way to measure interactions between factors affecting health is to use the Social Ecological Model (SEM) (12). This model studies how the physical, social, and cultural dimensions and political environments of the individual, as well as their personal characteristics, influence health, well-being, and social cohesion. The SEM recognizes interactions across individuals embedded within larger social systems and describes the characteristics of individuals and environments that underlie health outcomes. In the SEM, each level overlaps with other levels. Hence, defining the best public health strategies requires that a wide range of perspectives be targeted.

Although our purpose here is not to investigate how to adapt the previously cited models to COVID-19, we emphasize the importance of accounting for multiple variables simultaneously with the perspective of complex adaptive systems (9, 13).

## Why Does the Management of Lockdown-Lifting and of an Endemic Infectious Disease Raise Questions for the Scientific Community and Public Health Actors? A Dynamic Perspective Minimizing Societal Impacts

Minimizing the societal impact inevitably leads to making trade-offs between various components and choices on how to allocate the resources to different societal functions. The trade-offs include, for instance, health and wealth, individual freedom and collective duty, child access to education and senior outdoor access, medical and non-essential activities, international market losses and long-term tax increases, and all of the multiple combinations of these items. This situation corresponds to an economic dynamic optimization problem under constraint in an uncertain and moving environment (14). The economic term is, of course, to be understood in its primary sense of resource allocation and wealth sharing and is not limited to its monetary component. Hence, the relevant question is how to design the best policy under constraints.

### Biological and Economic Constraints

The biological constraints linked to COVID-19 and lockdown-lifting are known and have been extensively studied under various situations (15–19). The constraints arise both from the characteristics of the outbreak (epidemiological parameters, i.e., contagiousness and severity of the disease) and from the structure of the healthcare system (number of available beds, testing facilities, personnel). It is primarily a question of defining the modalities of lockdown-lifting that will not saturate the healthcare system, which would lead to excess mortality due to lack of patient care (19).

In the context of lockdown-lifting and living with an endemic disease, the major economic constraints arise from business resumption and societal benefits. The brutal and general cessation of economic activities has been widely accepted in the case of COVID-19, as was the application of national solidarity for the most affected individuals. However, the prolongation of lockdown leads us to question both the cost-effectiveness of this policy and its acceptability to individuals. A cessation of activity also generates a steep increase in public expenses and simultaneously a decrease in revenue (taxes). This situation leads us to seek a compromise between the resumption of activities and public health. For each resurgence of COVID-19, the issue will re-appear in very similar ways.

The behavior of individuals and their compliance with the potential rules issued for lockdown-lifting represent a major economic component of lockdown-lifting. In the case of selective lift, some people will benefit from population protection, provided in part by the share of the population remaining locked (social benefit) and will also benefit from their private benefits (resumption of activity and freedom of movement). Locked-down people will benefit from protection by being unexposed, as well as from the social benefits derived from the lifting (the contribution to society of the workers), in return for respecting the lockdown. The former would benefit from the positive externality generated by the collective's restrictive measures without having to assume the private costs. However, such free-riding may significantly reduce the effectiveness of the policy, as frequently illustrated in other settings for public good or public health management (20–22).

### Biological and Economic Uncertainty

The COVID-19 crisis is an example of management in an uncertain context due to the novelty in biological terms (new virus) and economic terms (large-scale shock). It is not only a question of considering the risk (which is likely) but of uncertainty, which is associated with a higher level of lack of knowledge (we do not know and do not know how to predict). This high degree of uncertainty is often fairly misunderstood (or tolerated) by populations and stakeholders.

SARS-Cov2 is a new pathogenic agent, and therefore there several biological uncertainties exist regarding the detection and care of afflicted individuals and its population dynamics (23). Considerable efforts are underway around the world to strengthen the level of knowledge of its pathophysiology or therapy in order to better treat, cure, predict, and manage the behavior of the epidemic and its consequences on individuals. The fact remains that today, the lifting strategies must be defined with very uncertain parameters. For example, having vaccines available to a large population in the short term would allow for significantly different strategies than if the vaccine were only available on the shelf several years from now.

In parallel with biological uncertainties, at least two major economic uncertainties are identified. The first uncertainty relates to the costs and benefits of lockdown-lifting strategies and medium-term endemic COVID-19 management, which directly depend on the uncertainties of the economic impact of lockdown. The impact could be more or less significant depending on the type of shock that COVID-19 will represent (24). The negative economic impacts could be offset over a post-crisis period, with these benefits even potentially exceeding the losses, but these scenarios seem unlikely, given the intensity and globality of the crisis. The brutal, severe, and global cessation of economic activities suggests a major economic impact, at least in the mid-term, with pre-crisis activity levels not reached, at best, until 2021 (25).

The second economic uncertainty consists of the resilience of our social-ecological system and the possibility of renewal of our lifestyles. Interestingly, some western countries call for an exit from the present COVID-19 situation through an in-depth change of our societal growth model. As part of lockdown-lifting decision-making, it seems reasonable to target a hypothesis of a return to a “before pandemic” state, since this represents in the short term the main way to limit the impact of the crisis. It is moreover in these terms that the majority of economic impacts are measured to date, at least for 2020 (fall in GDP, tax revenues, etc.). Grounding the lockdown-lifting strategy on the current economic system does not exclude the development of alternative economic models in the long term.

## Toward a Holistic Approach of Population Health for the COVID-19 Crisis: the Four Principles

We propose to apply four main principles in the short and medium terms to manage COVID-19 lockdown-lifting and the following endemic disease situation. Importantly, these four principles focus on short-term policymaking based on available information and use real-time forecasts to adjust the strategies in the midterm. As for medicine, the rationale is to establish an evidence-based policy intervention following a diagnostic relying on a systemic evaluation of a set of information (observations, data, tests, previous knowledge).

### Principle 1: Integrate Multiple and Heterogeneous Information to Diagnose and Act With Accuracy

There is an exponentially increasing amount of information available on the COVID-19 situation. The critical information required for rational decision-making is as yet still limited, and fake news and misinformation propagated massively via social networks blur evidence regarding public health management. There are major concerns about how to make quick decisions that combine up-to-date information. The limited rationality principles suggest the adoption of a procedural rationality approach, i.e., focusing on the process of how to make the best decision with an exponential availability of information rather than on trying to gather all the information, regarding its precision and relevance less. To address the paradox between data availability and its use in decision-making, multi-criterion analysis helps to gather data with various origins and combine information of different natures. It concatenates indicators and considers historical features, actor behavior, and expert opinions.

The integration of various information and metrics for an improved decision-making process may dramatically help to reach optimal societal benefits through balanced and equitable decisions. Epidemiological and economic modeling provide a set of highly valuable sources of information to consider in the holistic decision-making process. The holistic approach proposed and focusing on procedural rationality instead of substantial rationality are required all the more given that all the processes take place in a context of high uncertainty.

### Principle 2: Navigate With Uncertain Information, and Communicate It to the Population

COVID-19 requires that decisions be made under uncertainty, as we cannot predict the odds of some epidemiologic, economic, or political events occurring. For instance, it means implementing lockdown-lifting with neither precise information on the seroprevalence in the population and the distribution of seropositive individuals within the different subpopulations at risk (infants, adults, seniors…) nor on the location of contagious people. The nature of the contacts and the observance of biosecurity measures are complex and inconstant. People's behavior is changing (as the system changes), and the resilience of the economic system is not known. Computer simulations must be taken into account, as regular updates will provide new information improving the strategies adopted, but they also face uncertainty.

Uncertainty leads to dynamic adjustments of the decision, and the best decision today may not be the best tomorrow. This means that the decision process is based on biased information and that we must be clear on this within the communication strategy. Political path dependency, i.e., the tendency to keep the same policy, even if not really well-adapted anymore, so as to avoid any criticism on initial lack of vision, should clearly be avoided here, and the dynamics in political decisions should be highlighted and claimed as positive.

### Principle 3: Adjust the Strategies Dynamically

A feedback mechanism of the effectiveness of the measures taken allows us to continuously adjust the biological and economic dynamics and therefore represents a fine and precise regulatory tool if used and understood as such. Many metrics can be used for feedback. In animal medicine, feedback has been applied with success for decades for population health-related decision making. It is based, for instance, on clinical observations and on advanced health indicators provided by production and health data analyzed using machine learning tools (26). In the case of COVID-19, it would be a question of checking on a regular basis whether the predicted event really occurs and whether the trajectory is respected or deviated from. A comparison with the forecasts associated with the containment strategy would enable policymakers to relax or tighten certain rules. Unfortunately, the use of feedback in the management of the epidemic is often limited. On the one hand, the information used must be sufficiently reliable to support the decision, which is generally the case for the prevalence criteria, at least in the hospital system. On the other hand, the adjustment of the measures implemented in the management of outbreaks contradicts the path-dependence of the previous decision, to which policies are particularly sensitive, and requires a significant educational effort to be accepted.

Using the feedback principle for the COVID-19 situation appears a promising approach combining pragmatism and efficiency that will enable a precision health management approach at the local level (town, department) in accordance and complementarity with national rules. The success of its application in animal health population medicine can be duplicated for COVID-19.

### Principle 4: Manage Clusters With a Multi-Scalar Spatialized Policy

A multi-scalar policy of lockdown-lifting and endemic COVID-19 management will differentiate the strategies to be implemented as a function of the subpopulations and the ecosystem in which they live. Multi-scalar means well-integrated and coordinated multilevel policies. These principles are well-integrated into the epidemiological approaches of COVID-19 but not yet in the economic ones, whether it be the behavior of actors or their contributions to the creation of wealth.

This differentiated approach by cluster not only improves the performance of the policies for limiting the spread of the disease but it also integrates the interest, for individuals and groups, in unlocking certain populations gradually (27). Clustering allows the inclusion of equity instead of equality. Because collective and superior interest should prevail, applying equity means defining precise rules accounting for all subpopulations' well-being, the contributions of individuals to the collective value creation, and individual constraints at both the personal or familial and professional point of view. Seniors and the unemployed should have access to public areas, respecting given restrictions. Priority for freedom of circulation should remain for medical staff and their support as well as essential sectors. People performing partial home working without any drop in productivity should continue to do so in spite of some preferring to work only at the office. On the contrary, people with a low socio-professional level and no possibility of working at home should be authorized to contribute whatever they can to global societal value. Combining epidemiological clustering with outbreak management and economic clustering though the contribution of socio-professional groups to societal value may help to achieve the best societal benefit.

In the context of COVID-19, coupling the principle of feedback to a multi-scalar approach, at least with a segmented geographic approach, would make it possible to respond efficiently, and clarifying a precision approach—differentiated geographically and by population—would be facilitated.

## Implication: Operational Framework for Evidence-Based Policy Interventions for COVID-19

**Figure 1:**
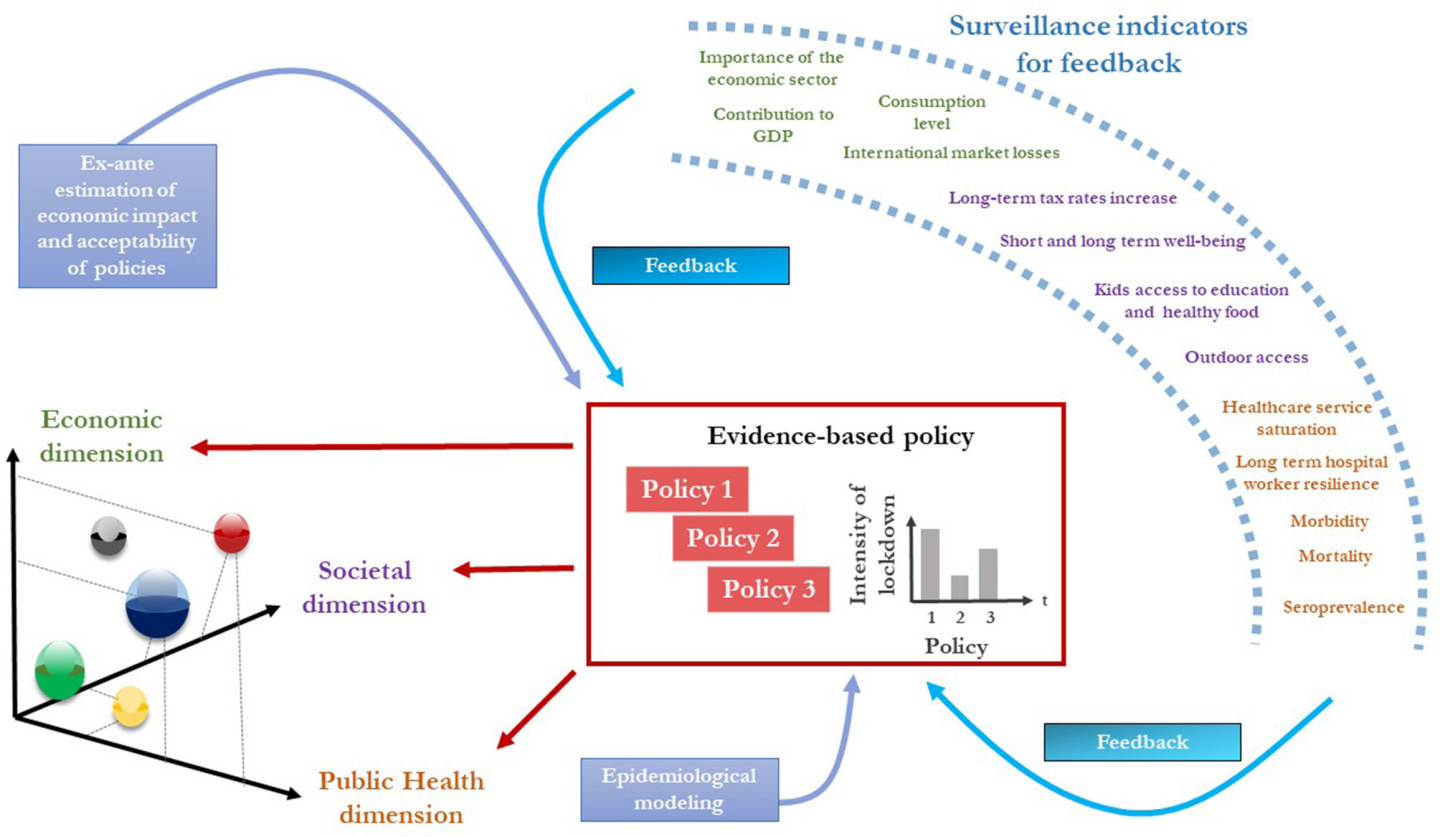
Framework for an evidence-based policy intervention for COVID-19. The central rectangle represents the successive evidence-based policies for COVID-19, supported by *ex-ante* epidemiological and economic modeling. The policy accounts for economic, societal, and public health dimensions (left) for each subpopulation (bubble), which are infants, adults, seniors, and the different socio-professional groups. Feedback mechanisms, based on surveillance indicators, help to precisely and promptly monitor and adjust the policy.

Because lockdown has increased social pressure, there might be strong protests against the different strategies to be adopted, all the more so the longer the lockdown and crisis last. Avoiding a political shock is a key point for policymakers but also for overall societal benefit. A “yellow-vest”-like crisis during COVID-19 management may have dramatic consequences. Considering the constraints encountered and the principles described above, we propose an evidence-based policy framework to handle the COVID-19 situation, as is applied to perform medical diagnostics for diseased patients (Figure 1). Any policy should envision (i) respecting an equilibrium among the three dimensions detailed below (public health, economics, and wellbeing), (ii) quantitatively, qualitatively, and continuously monitoring the societal-economic-epidemiological dynamics, allowing (iii) the policy to be adjusted by tightening or loosening measures before the epidemic damage may (re-)occur. The figure represents three successive policies implemented according to the proposed framework.

The framework includes scientific background from epidemiological and economic modeling readily available (blue boxes, Figure 1). The epidemiological transmission models used should consider the sub-populations in terms of biological risk (children, adults, seniors) as well as in terms of economic (socio-professional profiles) and political (socially vulnerable populations) impact. Epidemiological and bio-economic modeling are not a substitute for managing uncertainty, but they provide practical support for the expected results of each strategy, which can then be integrated into the overall decision-making process. A well-integrated and coordinated multilevel policy will differentiate the strategies to be implemented as a function of the subpopulations and their social-ecological system. Considering the subpopulations allows societal dimension of the issue to be practically accounted for (Figure 1), i.e., accounting for socio-professional categories (for their contribution to the collective production and their vulnerability), hard-to-reach populations (refugees, homeless, high precarious…), and long-term consequences (child and student education, reintegration of the unemployed…). Considering several social gradients guarantees a precision approach. A high-precision geographically differentiated strategy is possible, providing a high level of coordination of decision-makers within and between geographical areas.

Based on bio-economic modeling, an evidence-based policy can be implemented through the societal, economic, and public health dimensions, differently for various subpopulations. Importantly, the policy is not only the compromise of the monetary and public health dimensions but accounts for societal outcomes as well. Societal indicators refer to strategies that specifically consider non-active subpopulations, or subpopulations that do not directly contribute to monetary value production (GDP). The sub-population epidemiological modeling allows the strategy to be adjusted for minorities as well as for people with specific risk factors. Because COVID-19 is likely to become endemic, these social and societal well-being criteria (non-monetary economic) are key criteria to be accounted for. Policy 1 represents, for instance, a highly intensive level of lockdown (i.e., strict lockdown, as observed in many countries) that leads to strong negative economic and societal impacts by enhancing the public health dimension.

The feedback system guides a practical approach to manage uncertainty. A set of quantitative and qualitative indicators are proposed to precisely monitor the societal-economic-epidemiological dynamics (Figure 1, right par). It could, for instance, be based on active surveillance devices (tracking time or location depending on socio-professional profiles) implemented for alternate access to public areas for various populations at risk. Social criteria metrics such as real outdoor access could be used (i) to control abuses and to predict epidemiological metrics for the next week but also (ii) to evaluate how a well-being measure (outside access for the elderly, for instance) is welcome and adopted (policy evaluation). Such metrics help in measuring the needs and behaviors of the population and adapting the strategy. Epidemiological criteria such as mortality, morbidity, and the possible saturation rates of hospital and intensive care services could be used. The feedback overtakes the regular updating of the bioeconomics and epidemiological models that support decision-making and clearly and directly bridges the gap between the situation in the field and the situation as seen by policymakers. The early balancing process allows tightening or loosening measures before the epidemic damage (re-)occurs. Applying the feedback principle leads, for instance, to changing policy 1 into policy 2 at time 2 to balance the three dimensions and give room to breathe to economics and the locked-down population; for example, the surveillance indicators might show that policy 1 was efficient for outbreak control (decrease in morbidity and mortality and healthcare services no longer saturated), but that social (mental health, acceptability of the lockdown principle) and economic (bankruptcy, GDP decrease) indicators had reached a critical level. A few weeks later, a policy adjustment (policy 3) may occur, due to an increase in mortality and morbidity and a high level of healthcare service saturation), leading to limits being placed on outdoor access for populations with low contributions to the country's economic life (seniors, the unemployed, children), through a spatial-temporal sharing of public areas.

Altogether, this allows an evidence-based policy that steers the strategy with precision and avoids any political shock. Adapting the framework regionally would likely improve the efficiency of such a precision approach.

## Author Contributions

DR and GL conceived and wrote the paper and the figure. DR and GL contributed equally to the article, and approved the submitted version.

## Conflict of Interest

The authors declare that the research was conducted in the absence of any commercial or financial relationships that could be construed as a potential conflict of interest.
